# IMPACT OF RELIGION ON FRAMING AND CARING FOR DEMENTIA AMONG MUSLIM ARABS IN QATAR: A QUALITATIVE STUDY

**DOI:** 10.1093/geroni/igae098.0153

**Published:** 2024-12-31

**Authors:** Suhad Daher-Nashif, Suzanne Hammad, Tanya Kane

**Affiliations:** Keele University, Newcastle, England, United Kingdom; North Western Qatar, Doha, Ad Dawhah, Qatar; Qatar University, Doha, Ad Dawhah, Qatar

## Abstract

Studies have examined how religion plays a major role in framing mental disorders and in shaping caregiving practices. This presentation sheds light on dementia care among Muslim Arabs in Qatar. The presentation will examine how old age and dementia are framed in the Islamic religious resources, i.e., the Quran and Sunnah Nabawiyyah (statements and behaviours of Prophet Muhammad). Then it explores how these texts feed into the belief systems distributed within the community by sharia scholars, and later uncovers how these both sources are transformed to social behaviours in caring for older adults with dementia. The study uses descriptive qualitative methodology and is based on three data sources: Islamic religious texts i.e., the Quran and Sunnah, eight semi-structured interviews with Sharia scholars, and semi-structured interviews with 37 Arab-Muslim families from different Arab countries living in the state of Qatar. Thematic analysis has been applied to analyse the texts and the interviews. Compassion and honouring parents are commandments in the Islamic holy texts. The Quran and Hadith include definition and characteristics of ageing, of cognitive deterioration and give clear instructions on how and why older adults must be cared for. These values are expressed in the beliefs system constructed by sharia scholars that play a major role as motivators to caring for dementia patients by the families. Families used religious practices as diagnostic and treatment tools. Gaps between the holy text and the social beliefs were found in explaining dementia and Alzheimer. Moreover, religious practices played role in coping with caregiving-burden.

